# The Integration of Data from Different Long-Read Sequencing Platforms Enhances Proteoform Characterization in Arabidopsis

**DOI:** 10.3390/plants12030511

**Published:** 2023-01-22

**Authors:** Lara García-Campa, Luis Valledor, Jesús Pascual

**Affiliations:** 1 Plant Physiology, Department of Organisms and Systems Biology, University of Oviedo, 33003 Oviedo, Spain; 2 University Institute of Biotechnology of Asturias, University of Oviedo, 33003 Oviedo, Spain

**Keywords:** proteogenomics, long-read, sequencing, nanopore, PacBio, protein database, proteoform, ONT-DRS, Iso-Seq

## Abstract

The increasing availability of massive omics data requires improving the quality of reference databases and their annotations. The combination of full-length isoform sequencing (Iso-Seq) with short-read transcriptomics and proteomics has been successfully used for increasing proteoform characterization, which is a main ongoing goal in biology. However, the potential of including Oxford Nanopore Technologies Direct RNA Sequencing (ONT-DRS) data has not been explored. In this paper, we analyzed the impact of combining Iso-Seq- and ONT-DRS-derived data on the identification of proteoforms in Arabidopsis MS proteomics data. To this end, we selected a proteomics dataset corresponding to senescent leaves and we performed protein searches using three different protein databases: AtRTD2 and AtRTD3, built from the homonymous transcriptomes, regarded as the most complete and up-to-date available for the species; and a custom hybrid database combining AtRTD3 with publicly available ONT-DRS transcriptomics data generated from Arabidopsis leaves. Our results show that the inclusion and combination of long-read sequencing data from Iso-Seq and ONT-DRS into a proteogenomic workflow enhances proteoform characterization and discovery in bottom-up proteomics studies. This represents a great opportunity to further investigate biological systems at an unprecedented scale, although it brings challenges to current protein searching algorithms.

## 1. Introduction

Understanding how organisms work requires a holistic approach. Systems biology studies different layers of complexity of organisms in a high throughput manner, what we call omics, and then integrates them to obtain an accurate picture of an organism’s biology through its development or under different environmental conditions. Such an approach has been successfully applied in plant sciences to study, for example, the response to different types of stress in tree species [1,2,3,4,5,6,7], *Arabidopsis thaliana* [8,9] or *Chlamydomonas reinhardtii* [10,11]. However, the integration of different omic levels is a rather challenging task. It highly relies on the capacities of the analytical platforms and the quality of the reference databases (genomes, transcriptomes, proteomes) and their annotations. In this regard, the latest advances in sequencing technology can be useful to improve not only the quality of genomes and transcriptomes, but also the number of proteoforms, the different mature functional proteins produced from a single gene sequence, identified by proteogenomic approaches. Improving proteoform identification would increase the quality of the integration of transcriptomic and proteomic layers in systems biology approaches.

The latest breakthrough in sequencing technology, represented by long-read sequencing platforms, such as Pacific Biosciences (PacBio) and Oxford Nanopore Technologies (ONT), makes possible end-to-end sequencing of nucleic acid molecules. Such an increase in read lengths, in comparison to second-generation or next-generation sequencers, paves the way to address a large variety of research questions that have posed a challenge for short-read sequencing technologies. Longer reads allow sequencing through extended repetitive regions and mutations detection, which facilitates closing gaps in current reference genome assemblies and the characterization of structural variations. In general, it also makes it possible to generate high-quality whole-genome assemblies and increases the accuracy of gene annotation and isoform identification. PacBio and ONT sequencing have been successfully used for resolving and refining genome assemblies and expanding transcriptome characterization in a variety of species [12,13,14,15,16,17]. Long-read sequencing technologies have also been applied in diagnostics, and epigenetic and epitranscriptomic studies [18,19,20]. Therefore, the irruption of third-generation sequencers, with the possibility to obtain long, theoretically full-length reads is improving the quality of genome assemblies and transcriptomes.

The number of studies using Iso-Seq (full-length isoform sequencing) and ONT-DRS (direct RNA sequencing) are still relatively limited in plants, especially in the case of ONT-DRS, but they are steadily increasing, at least in the main research species, such as crops or the model species *A. thaliana* (hereafter Arabidopsis). In Arabidopsis, the number of new genes combined with the refinement and the discovery of new isoforms is impressive. The last reference transcriptome of this species (AtRTD3) contains twice the number of transcripts of the previous version (AtRTD2) [17]. More than 75% of those transcripts are from Iso-Seq. Similarly, ONT-DRS of different Arabidopsis developmental stages identified more than 38,500 novel transcript isoforms [13]. These studies are proof of the power of third-generation sequencing technologies to define new isoforms and splice variants and reveal a substantial underestimation of the complexity of Arabidopsis transcriptomes. This improvement in transcriptomes depth and quality can benefit proteome coverage in proteomic studies by enhancing the characterization of proteoforms. Protein identification in MS-based bottom-up proteomics relies on so-called searching algorithms that compare experimentally acquired MS spectra with theoretical spectra obtained through in silico digestion of the proteins in a protein/peptide database [21,22]. The importance of the database in this strategy is such that proteins that are not included in the database are simply not identifiable. Therefore, the completeness and quality of the database has a huge impact in proteome characterization [23,24]. In this context, high-quality transcriptomes generated by long-read sequencing can be a powerful tool to increase the number of proteoforms identified in proteomic studies, helping to improve the depth of the analysis and enhancing the potential for new discoveries. This type of approach, known as long-read proteogenomics as it is based on the use of databases generated from, or including, long-read RNA sequencing information against proteomics data, is a valid and powerful strategy to validate the existence of novel transcript isoforms at protein level. This type of long-read proteogenomic approach is relevant not only to continue refining genomes, transcriptomes and annotation models, but to improve the output of integrative systems biology approaches and thereby our understanding of living organisms. Such an approach has been successfully applied in humans [25], rice [26] and Arabidopsis [27,28], but only with Iso-Seq data and using transcriptomics and proteomics data generated from the same sample. The potential of including ONT-DRS data and of using databases built from data from different samples and sources has not been explored.

In this paper, we explore the impact of combining Iso-Seq- and ONT-DRS-derived transcriptomics data on the identification of proteoforms in MS proteomics data in Arabidopsis. This species was selected because of the public availability of datasets covering proteome and third generation sequencing. An Arabidopsis proteomic dataset, part of the mass-spectrometry-based draft of the Arabidopsis proteome, was selected for its coverage and the use of state-of-the-art mass spectrometry instrumentation for its generation [29]. We selected the proteomics data corresponding to senescent leaves, as this developmental stage can be triggered by different types of stress and it partially resembles stress [30], which is currently one of the main topics of interest in plant sciences and one of the main topics of study using systems biology approaches. The Arabidopsis senescent leaves proteomic data were analyzed using two different versions of the Arabidopsis reference transcriptome, AtRTD2 and AtRTD3, the latter largely based on Iso-Seq-derived high-confidence transcripts, and a custom hybrid database combining AtRTD3 with publicly available ONT-DRS transcriptomics data generated from Arabidopsis leaves. Our results show that Iso-Seq and ONT-DRS transcriptomic data are useful to validate and discover new proteoforms associated with senescence in Arabidopsis, and how the different sequencing technologies can be combined for a better proteome characterization. We also discuss some limitations we encountered and the challenges long-read proteogenomics faces.

## 2. Results

### 2.1. The Different Protein Databases Represent Complementary Arabidopsis Proteomes

**Figure 1:**
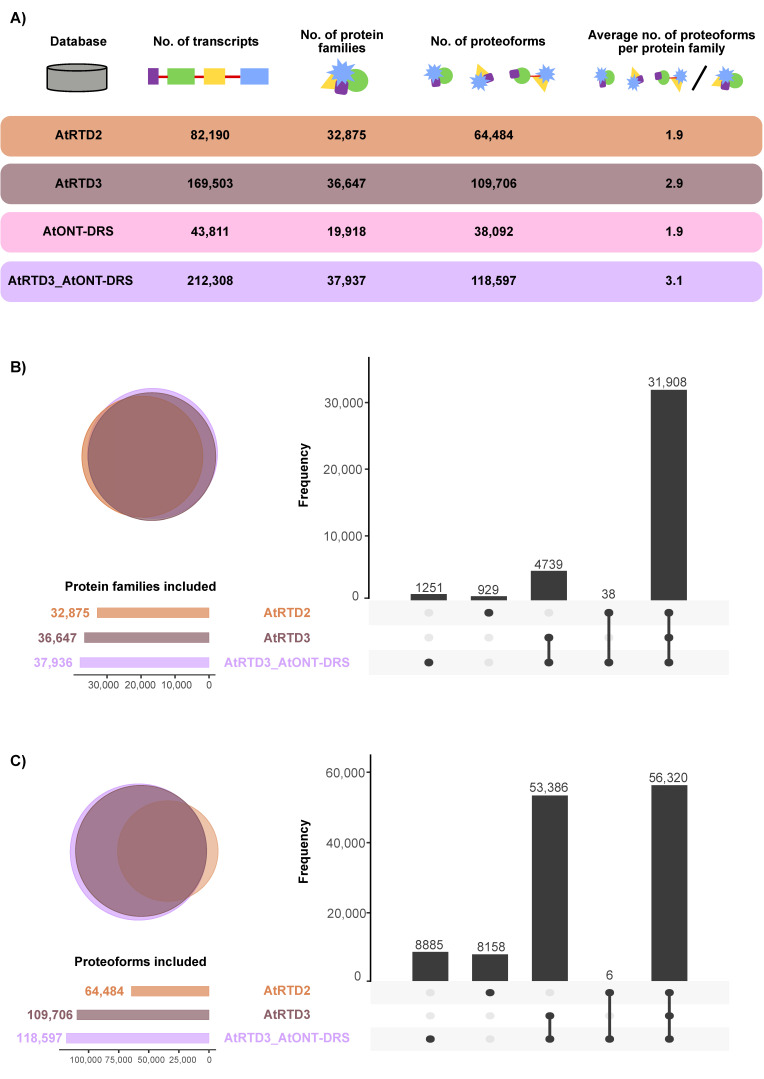
Overview of the different protein databases used in this study. (**A**) Number of transcripts, proteoforms and average proteoforms per protein family in each database. (**B**) Venn diagram and UpSet plot showing the overlap between the protein families included in the different protein databases used in this study. (**C**) Venn diagram and UpSet plot showing the overlap between the proteoforms included in the different protein databases used in this study.

The two last versions of the *Arabidopsis thaliana* Reference Transcript Dataset (AtRTD2 and AtRTD3) were selected as the most accurate and comprehensive transcriptomes available for this species [17,31]. AtRTD2 includes the original AtRTD1 [32], generated from the merge of transcripts from TAIR10 and from alternative splicing discovery analysis, merged with Araport 11 and a collection of RNA-Seq samples corresponding to different tissues, developmental stages, and treatments, which was filtered to remove redundancies. AtRTD3 was, in turn, built by appending a revised-for-artifacts version of AtRTD2 to an Iso-Seq-based high confidence transcriptome generated from a compendium of samples from different tissues and experimental conditions. In addition to AtRTD2 and AtRTD3, a custom high-confidence transcriptome, based on ONT-DRS data, was constructed in-house (AtONT-DRS) to include in the study the other main long-read sequencing technology available in the market now, along with Iso-Seq. The selected ONT-DRS data were generated from 14-day-old plants grown on MS plates [19]. AtONT-DRS contained 43,811 non-redundant transcripts (Figure 1A, Supplemental Data S1). In comparison, AtRTD2 contained 82,190 and AtRTD3 169,503 transcripts (Figure 1A, Supplemental Data S2 and S3).

The three reference transcriptomes were translated into proteins using TranSuite, an algorithm-based ORF identification translation tool. After removing duplicated proteins, AtRTD2 contained 64,484 proteoforms, 1.9 per protein family; AtRTD3 contained 109,706, 2.9 per protein family; and AtONT-DRS 38092, 1.9 per protein family (Figure 1A, Supplemental Data S1–S3). To avoid misleading interpretations on the potential of AtONT-DRS to identify proteoforms due to its lower complexity, considering the principle of parsimony applied by protein searching algorithms, and to filter out proteoforms already included in AtRTD3, we appended AtONT-DRS to AtRTD3 (AtRTD3_AtONT-DRS) for protein searches (Supplemental Data S4). In the cases of protein duplication, the protein accession of the protein in AtRTD3 was maintained. AtRTD3_AtONT-DRS contained 212,308 transcripts representing 118,597 proteoforms belonging to 37,937 protein families, which makes up 3.1 proteoforms per protein family (Figure 1A). Therefore, AtONT-DRS added a significant number of proteoforms compared to AtRTD3, although it contained comparably less as it was generated from just one type of sample from a single experiment.

The proteoforms in the different databases represented a different number of protein families in each case as a consequence of the different nature of the samples and experiments of the sequencing data each database was generated from. The proteoforms in AtRTD2 and AtRTD3 represent more than 32,000 and 36,000 protein families, respectively (Figure 1A). The coverage was smaller in AtONT-DRS (19918 protein families, Figure 1A), which was not surprising, as it was generated from sequencing data from a much less rich and diverse collection of samples in comparison to AtRTD2 and AtRTD3 due to the scarcity of ONT-DRS data in public repositories. However, its combination with AtRTD3 (AtRTD3_AtONT-DRS) covered more than 37,900 protein families (Figure 1A), including 1251 unique protein families, i.e., included in neither of the other two protein databases (Figure 1B). AtRTD2 also included unique protein families (Figure 1B). However, most of the protein families were represented in the three databases, while a lower number of them were common to only two of them (Figure 1B). Interestingly, 38 were common between AtRTD2 and AtRTD3_AtONT-DRS (Figure 1B). At proteoform level, the majority of proteoforms were included in the three databases, as expected (Figure 1C). Noteworthy, AtRTD2 and AtRTD3_AtONT-DRS showed a remarkable number of database specific proteoforms (Figure 1C), coming in the case of the latter from AtONT-DRS. Therefore, they were novel proteoforms, i.e., they were not previously included in AtRTD3.

In summary, the different protein databases represented different Arabidopsis proteomes and contained different sets of proteoforms, including some potentially novel, overall representing partially complementary proteomes. Remarkably, the transcriptomic data generated by ONT-DRS provided an increase in richness and complexity at the protein level that exceeded that expected from its relative simplicity and smaller size, as it was the result of a single sequencing experiment, not a compendium of tissues and situations, such as AtRTD2 and AtRTD3.

### 2.2. The Inclusion of Iso-Seq- and ONT-DRS-Derived Data in Protein Databases Enhanced the Characterization of Proteoforms in Proteomics Data

AtRTD2, AtRTD3 and AtRTD3_AtONT-DRS were used for protein searching in a set of proteomics data consisting of 24 fractions from Arabidopsis senescent leaves, part of the mass-spectrometry-based draft of the Arabidopsis proteome [29]. The reasoning behind the selection of the proteomics data was to select a dataset from an experiment and an analytical approach designed to maximize proteome coverage, i.e., generated with state-of-the-art instrumentation, an Orbitrap Lumos mass spectrometer in this case, and including sample fractionation approaches.

**Figure 2:**
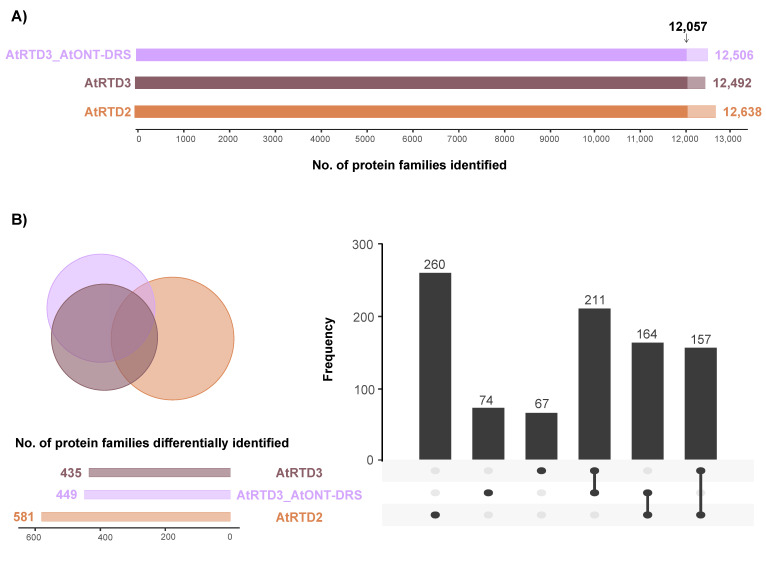
Identification of protein families in proteomics data from senescent leaves in Arabidopsis. (**A**) Total number of protein families identified with each database. The arrow marks the protein families identified in common by the three databases. (**B**) Venn diagram and UpSet plot showing the overlap between the protein families identified with each database, excluding the 12,057 families identified by the three of them.

The three databases identified a similar number of protein families (Figure 2A; Supplemental Tables S1–S3) with an overlap of 12,057 proteins between them (Figure 2A). Interestingly, 260 proteins were exclusively identified using AtRTD2 and 67 with AtRTD3_AtONT-DRS (Figure 2B). Noteworthy, AtRTD3 is not AtRTD2 merged with Iso-Seq data, but a refinement of AtRTD2 was performed previously to remove artifacts, and AtONT-DRS was appended to AtRTD3 (AtRTD3_AtONT-DRS). Consequently, the databases covered different parts of the genome and the proteome, as it has been detailed in the previous section (Figure 1). Therefore, the identification of several database-specific proteins was not surprising, and it was expected [33].

**Figure 3:**
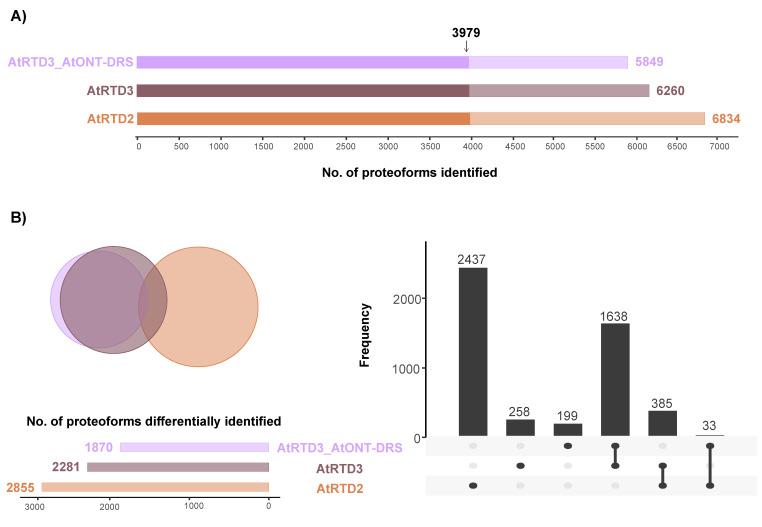
Proteoform identification in a MS proteomics dataset corresponding to Arabidopsis senescent leaves. (**A**) Total number of proteoforms unambiguously identified with each database. The arrow marks the proteoforms that were identified by the three databases. (**B**) Venn diagram and UpSet plot showing the overlap between the proteoforms identified with each database, excluding the 3979 identified by the three of them.

At proteoform level, focusing on unambiguously identified isoforms, i.e., proteoforms identified with at least one unique peptide, the number of identifications decreased along with the increasing number of proteoforms in the database (Figure 3A). We found a large overlap of 3979 proteoforms between the three databases (Figure 3A). In addition, there was a significant overlap between AtRTD3 and AtRTD3_AtONT-DRS, which was not surprising considering how the latter was built (Figure 3B). The intersection between AtRTD2 and AtRTD3 included 385 proteoforms (Figure 3B). Moreover, 2437 proteoforms were identified with AtRTD2 only, 258 with AtRTD3 and 199 with AtRTD3_AtONT-DRS (Figure 3B).

Regarding the high number of protein families and proteoforms identified with AtRTD2, a closer analysis of the identifications revealed a quite complex scenario. In some cases, a protein family included in AtRTD2 was not present in AtRTD3 or AtRTD3_AtONT-DRS. However, these were the least and a vast majority of proteoforms identified only with AtRTD2 corresponded to protein families with a lower number of proteoforms in AtRTD2 in comparison with AtRTD3 and AtRTD3_AtONT-DRS; this changed the profile of unique peptides completely, with many unique peptides in AtRTD2 not maintaining that condition in AtRTD3 or AtRTD3_AtONT-DRS (Supplemental Table S4). The same was observed between AtRTD3 and AtRTD3_AtONT-DRS, although at a smaller magnitude (Supplemental Table S5). In other cases, the same proteoform had a slightly different sequence in AtRTD2 compared to AtRTD3, which is a consequence of the refinement performed to build AtRTD3. Further inspection of the peptides identified with AtRTD2 and their assignment to proteoforms and the comparison with those identified with AtRTD3_AtONT-DRS revealed that the peptides assigned to a given proteoform in AtRTD2 in some cases were assigned either to another proteoform group or to another proteoform very similar in protein sequence but belonging to a different protein family when using AtRTD3_AtONT-DRS (Supplemental Table S4). This observation evidenced some annotation inconsistencies between the tested database releases. In other cases, some peptides identified using AtRTD2 were simply not identified when performing the search with AtRTD3 or AtRTD3_AtONT-DRS (Supplemental Table S4). This might be the consequence of differences in peptide scoring and identification confidence derived from the different set of proteoforms and resulting decoys generated from each database during protein searches. This might also be the case for some proteoforms differentially identified between AtRTD3 and AtRTD3_AtONT-DRS (Supplemental Table S5).

As for the number of proteoforms identified per gene, no more than three proteoforms per protein family were identified with AtRTD2, while that number increased to 11 in the case of AtRTD3 and to 8 when performing the identification using AtRTD3_AtONT-DRS as the reference database (Supplemental Tables S1–S3). On average, AtRTD3 and AtRTD3_AtONT-DRS accomplished a 2% increase in the number of identified proteoforms per gene.

### 2.3. The Inclusion of Iso-Seq and ONT-DRS Sequencing Data Allowed Identifying a Higuer Number of Proteoforms Associated to Leaf Senescence

The proteomics data used in this study were generated from Arabidopsis senescent leaves. To further explore the potential of including PacBio Iso-Seq and ONT-DRS transcriptomics data in protein databases for the discovery of new proteoforms, we followed a combined approach. First, we performed differential gene expression analysis on sample-matched RNA-Seq data generated in the same study as the proteomics data we used in the protein searches [29]. We found 4277 differentially expressed genes (log_2_FC > |1.5|, adjusted *p*-value < 0.05; Supplemental Table S7). At least one proteoform coming from Iso-Seq or ONT-DRS was identified for 331 of them (Supplemental Table S6), including 26 novel forms obtained from ONT-DRS (Supplemental Table S6), 8 of which are, in addition, included in the Leaf Senescence DataBase 4.0 (LSD 4.0) [34], which collects the knowledge on leaf senescence, including the genes involved. A further examination of our list of proteoforms identified from long-read-derived protein data revealed 30 more new ONT-DRS proteoforms belonging to protein families included in LSD 4.0 (Supplemental Table S6). These included ASPARTATE AMINOTRANSFERASE 5 (ASP5; AT4G31990), HIGH CYCLIC ELECTRON FLOW 1 (HCEF1; AT3G54050), AMP-DEPENDENT SYNTHETASE AND LIGASE FAMILY PROTEIN (AA3; AT3G48990), ACONITASE 1 (ACO1; AT4G35830), CATALASE 3 (CAT3; AT1G20620) and CYTRATE SYNTHASE (CSY3; AT2G42790) (Supplemental Table S6). In addition, 93 Iso-Seq proteoforms from 76 protein families, included in AtRTD3, were associated with leaf senescence according to LSD 4.0 (Supplemental Table S6), such as GLUTAMINE-DEPENDENT ASPARAGINE SYNTHASE 1 (ASN1; AT3G47340), upregulated in senescent leaves and its expression suggested to be associated with cell sugar levels [35]. Interestingly, we found Iso-Seq proteoforms from protein families for which we identified proteoforms that came from AtONT-DRS, demonstrating the potential of our combined long-read proteogenomic approach. These included ACO1, CSY3 and CAT3.

**Figure 4:**
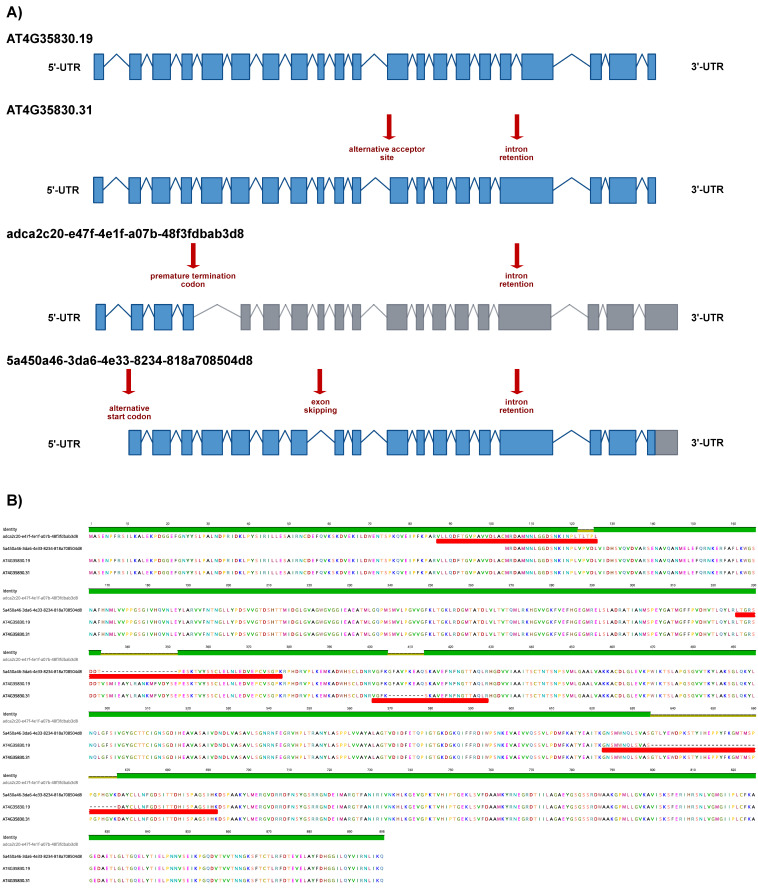
ACONITASE 1 proteoforms characterization in Arabidopsis senescent leaves employing a combined long-read proteogenomic approach. (**A**) Schematic representation of the ACONITASE 1 (ACO1) transcript isoforms corresponding to the proteoforms identified in the protein search performed with the AtRTD3_AtONT-DRS protein database. Boxes represent exons and lines represent introns. Exons colored in blue are translated, while exons in grey are not according to the protein translation prediction performed with TranSuite. Red arrows highlight characteristic alternative splicing- and coding-related features in each transcript isoform. (**B**) Alignment of the ACO1 proteoforms identified in the protein search performed with AtRTD3_AtONT-DRS. Red boxes indicate the unique peptides identified in each case. The identity graph displays the degree of identity across all sequences for every position. Green means that the residue at the position is the same across all sequences. Positions with 30% to under 100% identity are represented in yellow.

**Figure 5:**
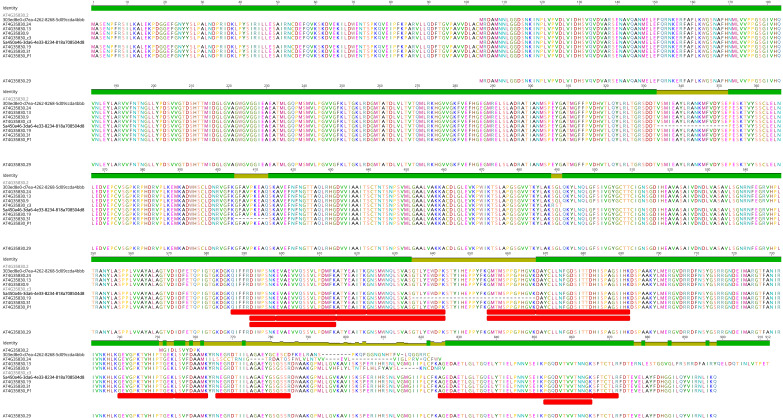
Alignment of the AtRDT2 ACONITASE 1 (ACO1) proteoform AT4G35830_P1 and the ACO1 proteoforms included in AtRTD3_AtONT-DRS containing the peptides assigned as unique peptides to AT4G35830_P1 (red boxes) when performing the protein search using AtRTD2 as the reference protein database. The identity graph displays the degree of identity across all sequences for every position. Green means that the residue at the position is the same across all sequences. Positions with 30% to under 100% identity are represented in yellow.

As a representative example of the obtained results, we show ACO1 in detail. AtRTD3_AtONT-DRS contained 26 ACO1 proteoforms (Supplemental Data S4). We identified four of them in the senescent leaves’ proteomics data: 5a450a46-3da6-4e33-8234-818a708504d8 and adca2c20-e47f-4e1f-a07b-48f3fdbab3d8 coming from AtONT-DRS, and AT4G35830.19 and AT4G35830.31 from Iso-Seq. The four proteoforms are originated from four transcript isoforms with different exon chains derived from different alternative splicing events and with different predicted coding-related features, as a premature termination codon (PTC) and, consequently, a long 3′-UTR in adca2c20-e47f-4e1f-a07b-48f3fdbab3d8 (Figure 4A). These transcript isoforms result in four different ACO1 proteoforms, identified by at least one unique peptide (Figure 4B). Interestingly, we identified that a unique peptide from adca2c20-e47f-4e1f-a07b-48f3fdbab3d8, despite its transcript counterpart, contains a PTC, making it a potential target for degradation through the nonsense-mediated decay pathway (NMD) [36,37]. In comparison, AtRTD2 included just three ACO1 proteoforms (Supplemental Data S2). The protein search performed with AtRTD2 identified only one ACO1 proteoform: AT4G35830_P1, renamed as AT4G35830.4 in AtRTD3. This proteoform was, however, not identified when proteins were searched against the more complex AtRTD3_AtONT-DRS protein database. The ultimate reason for this was that the identification of AT4G35830_P1 with AtRTD2 was based on a unique peptide that lost its uniqueness in AtRTD3_AtONT-DRS due to the increased number of ACO1 proteoforms derived from the inclusion of Iso-Seq and ONT-DRS data. Those peptides were also present in 5a450a46-3da6-4e33-8234-818a708504d8, 303ed8e0-d7ea-4262-8268-5d09ccda4bbb, AT4G35830.9, AT4G35830.13, AT4G35830.19, AT4G35830.24 AT4G35830.29 and AT4G35830.31 in AtRTD3_AtONT-DRS (Figure 5, Supplemental Tables S5 and S8–S10).

In summary, the inclusion of protein information derived from third-generation sequencing allowed identifying new proteoforms associated with leaf senescence. Furthermore, it improved proteoform identification accuracy by increasing their coverage in the reference proteome. Notably, Iso-Seq and ONT-DRS data proved to complement each other.

## 3. Discussion

The ability to produce multiple proteoforms from a single gene represents an efficient tool for diversifying proteins functions and has an impact over plant physiology, including development and stress response [38,39]. Exploring the depth, significance, and potential of this ability to increase proteome complexity is a main ongoing goal in biology. Proteoforms can have, among others, differential biological functions, expression patterns, cellular localizations, or interaction partners. Proteoforms can arise from posttranscriptional modifications of a single pre-mRNA by alternative splicing or editing, and from posttranslational modifications (PTMs), either enzymatically catalyzed or spontaneous. Effective proteoform identification is a challenge and it is a main bottleneck to advance our knowledge on the role and relevance of different proteoforms [40]. In this study, we have used a proteogenomic approach to evaluate the impact of including protein information from third-generation sequencing transcriptomics data in protein databases over the identification of proteoforms derived from pre-mRNA posttranscriptional modifications in bottom-up proteomics data from Arabidopsis senescent leaves.

Protein identification is a central step in any bottom-up proteomics analysis workflow. Typically, protein identification relies on a protein database. This approach is usually referred to as “database searching” and it consists of correlating acquired fragment ion spectra with theoretical spectra predicted for each peptide contained in a protein sequence database [21,22]. Theoretical spectra are generated by in silico digestion of the protein database. The robustness of protein identification results following a database search approach is contingent upon the completeness and accuracy of the used reference protein database, including the annotations. Any protein not included in the database will never be identified. Therefore, the importance of the database is paramount for protein identification and there is a need for protein databases that are as exhaustive and accurate as possible, including annotations, as it has been shown in *C. reinhardtii* [33]. In this regard, proteogenomic approaches, combining the analysis of genomics or transcriptomics with proteomics, has been proved crucial to enhance the identification of proteoforms, including previously unknown proteins, in bottom-up proteomics by improving the quality of protein databases [41,42].

In Arabidopsis, the last version of the transcriptome, AtRTD3, used in this study, is largely based on Iso-Seq data generated from a collection of samples and situations, including different organs and plants exposed to different abiotic stresses or environmental cues [17]. AtRTD3 includes 169,503 transcripts, doubling the number of transcripts included in the previous version, AtRTD2 [17]. In comparison, our custom database generated from ONT-DRS data (AtONT-DRS) included a much lower number of transcripts (43,811) as it was generated from ONT-DRS data from a single experiment, instead of a compendium of situations and organs. However, it remarkably included 42,805 new transcript isoforms regarding AtRTD3, which was used as the reference transcriptome for the construction of AtONT-DRS. Those 42,805 new transcript isoforms yielded 29,201 non-redundant proteoforms when appended to AtRTD3 to obtain the hybrid AtRTD3_AtONT-DRS protein database, which indicates that most of the novel transcript isoforms from AtONT-DRS differed in their UTRs. Furthermore, the inclusion of ONT-DRS-derived data increased the number of proteoforms per protein family to 3.1 (Figure 1A), improving that of AtRTD3, regarded as the most updated and complete Arabidopsis transcriptome. Furthermore, AtONT-DRS contributed proteoforms from protein families not represented in AtRTD3 (Figure 1C). Therefore, it seems clear that ONT-DRS transcriptomics data have the potential to add further protein knowledge compared to that generated from Iso-Seq, complementing it, even in a case like this, in which ONT-DRS transcriptomics data from a single experiment were used. This is consistent with reported differences between Iso-Seq and ONT-DRS when it comes to read length and error rates, or the proportion of reads mapping to the reference genome in Arabidopsis [12]. Overall, this complementarity between both sequencing technologies suggests a potential to improve the output of long-read based proteogenomic approaches by combining them. Our results showed an improvement in the number of proteoforms identified per gene with AtRTD3_AtONT-DRS, the potential to identify new proteoforms (Figure 4) and, furthermore, to increase the accuracy of proteoform identifications (Figure 5), which allowed a better characterization of the Arabidopsis senescent leaves proteome.

Despite these clear advantages and improvements, we detected some limitations. The total number of proteoform and peptide identifications did not increase in concordance with the higher number of proteoforms included in the database. In fact, AtRTD3_AtONT-DRS was the database identifying the lowest number of proteoforms (Figure 3A). Similarly, AtRTD3 and AtRTD3_AtONT-DRS identified a lower number of peptides in comparison with AtRTD2 (Supplemental Tables S8–S10). This can be a consequence of the lack of sample-matched transcriptomics and proteomics data, i.e., the protein databases can include proteins that are not expressed in senescent leaves and vice versa. Such a contradictory performance has, however, been observed empirically in other long-read proteogenomic approaches [42,43]. Integrating long-read sequencing and proteomic data is challenging due to the increased complexity of transcriptomes and the large size of resulting protein databases. A higher number of proteoforms with a high number of shared peptides, which is a common feature in eukaryotes, poses a computational challenge for current searching algorithms, especially when it comes to protein inference, and it leads to lower-sensitivity peptide identifications [42], which might be the reason for some peptides being identified with AtRTD2 or AtRTD3, but not with the larger AtRTD3_AtONT-DRS protein database (Supplemental Tables S4 and S5). Several strategies have been proposed to overcome this limitation, including transcriptome-informed protein database reduction, sample-matched approaches, accurate and alternative FDR control strategies or the use of algorithm-based ORF prediction tools [42]. In addition, new searching algorithms dealing with this issue have started to be developed, such as *Rescue & Resolve*, which incorporates long-read transcript abundance information into the protein inference process [25]. However, no strategy has provided a fully satisfactory solution so far and how to maximize the number of identifications maintaining good reliability levels is an issue that remains largely unsolved.

Then, taking full advantage of long-read proteogenomics requires new computational development and the design of new algorithms and strategies aimed at dealing with increasingly complex protein databases. Nonetheless, the incorporation of long-read sequencing from both Iso-Seq and ONT-DRS into proteogenomics workflows has been proved to complement each other, increasing the potential to enhance the characterization of proteoforms in bottom-up proteomics studies, including the potential for discovering novel proteoforms in Arabidopsis. This type of approach could be applied to other sequenced species, being helpful to refine current proteogenomics results. This would provide, for example, a novel overview of the diversity and versatility of proteoforms underlying plant phenotypical responses to environmental stresses [38]. It also represents a tremendous opportunity for isoform-resolved investigations in translational research, since proteoforms have been long used in medicine as health and disease biomarkers [44]. This approach would also be beneficial for non-model species, for which a sequenced genome is not available. The combination of long-read platforms makes it possible to quickly generate high-quality databases, increasing the accuracy of gene annotations and isoform identifications, thus refining reference genomes and facilitating protein and proteoform identification. In summary, this represents a tremendous opportunity for advancing our understanding of the importance and the role of different proteoforms and of living organisms in general by boosting the potential of integrative systems biology approaches, although it brings new challenges along and it still has some limitations. 

## 4. Materials and Methods

### 4.1. Protein Databases Construction

AtRTD2 transcriptomic and annotation data were downloaded from https://ics.hutton.ac.uk/atRTD/. AtRTD3 transcriptomic, proteomic and annotation data were gathered from https://ics.hutton.ac.uk/atRTD/RTD3/.

ONT-DRS data corresponding to 14-day-old Arabidopsis plants growth on MS plates from Parker et al. 2021 were downloaded from the European Nucleotide Archive (accession code PRJEB32782). 

AtONT-DRS was built using Full-Length Alternative Isoform Analysis of RNA (FLAIR), a computational workflow specifically designed to correctly determine high-confidence transcripts and alternative splicing isoforms from ONT-DRS sequencing reads [45]. Settings by default were used. ONT-DRS files were processed and collapsed with FLAIR using AtRTD3 transcriptome and its annotation as a reference. 

Algorithm-assisted protein translation was performed with TranSuite [46]. Unlike traditional translation tools, TranSuite is not just restricted to find the longest ORF, but allows the identification of transcript CDSs, gene-level selection of transcription start sites, which leads to a more accurate translation of transcript isoforms, and the identification and characterization of different coding-related features, such as coding potential, similar-translation features or alternative ORFs.

Many transcript isoforms from the same gene differed in their UTRs sequences, but not in their CDS. Consequently, they resulted in the same protein. Therefore, it was necessary to remove redundant proteins (Supplemental Table S11, Supplemental Data S1–S4). Redundant sequences were identified and removed using rmdup, included in SeqKit v.2.3.1 [47].

### 4.2. Protein Identification

Proteomics mass spectrometry raw data from Arabidopsis senescent leaves, part of the mass spectrometry-based draft of the Arabidopsis proteome [29], were downloaded from PRIDE (project PXD013868). Protein searches were performed with MSFragger [48], included in the FragPipe suite, using the *Default* workflow. 

### 4.3. Differential Gene Expression Analysis

RNA-Seq raw data matching proteomics data from senescent Arabidopsis leaves were downloaded from ArrayExpress (www.ebi.ac.uk/arrayexpress, identifier E-MTAB-7978). Quality control was performed with FastQC and Trimmomatic [49] to perform adapters searching, clipping and quality trimming. Reads shorter than 31 bases after these processes were filtered out. RNA-Seq reads were pseudo-aligned to a custom in-house generated hybrid Arabidopsis transcriptome (AtRTD3_AtONT-DRS) and quantified with Salmon v.1.4.0 [50]. Differential gene expression analysis was performed with DESeq2 Bioconductor package v.1.30.1 [51] using a likelihood ratio test in R. Absolute log_2_FC > 1.5 and adjusted *p*-values < 0.05 were considered differential. The analysis was performed in R environment v.4.2.1 [52] run in rStudio v.2022.07.2 [53].

### 4.4. Protein Alignments

Protein alignments were performed in Geneious Prime v.2022.2.2 using Clustal Omega v.1.2.3 grouping sequences by similarity. 

### 4.5. Gene Model Plots

Gene model plots were generated according to gene annotations using the package *genemodel* v.1.1.0 in R environment v.4.2.1 [52], run in rStudio v.2022.07.2 [53]. 

## Data Availability

AtRTD2 transcriptomic and annotation data are available at https://ics.hutton.ac.uk/atRTD/. AtRTD3 transcriptomic, proteomic and annotation data are available at https://ics.hutton.ac.uk/atRTD/RTD3/. Arabidopsis senescent leaves proteomics raw data is available at PRIDE (https://www.ebi.ac.uk/pride/; project PXD013868) and the corresponding RNA-Seq raw data can be downloaded from ArrayExpress (www.ebi.ac.uk/arrayexpress, identifier E-MTAB-7978).

## Supplementary Materials

The following supporting information can be downloaded at: https://www.mdpi.com/article/10.3390/plants12030511/s1, Table S1: List of proteoforms identified using AtRTD2; Table S2: List of proteoforms identified using AtRTD3; Table S3: List of proteoforms identified using AtRTD3_AtONT-DRS; Table S4: Protein mapping of the peptides identified with AtRTD2 in comparison to their mapping when performing protein searching with AtRTD3_AtONT-DRS; Table S5: Protein mapping of the peptides identified with AtRTD3 in comparison to their mapping when performing protein searching with AtRTD3_AtONT-DRS; Table S6: List of new proteoforms identified and their involvement in leave senescence; Table S7: List of differentially expressed genes in Arabidopsis senescent leaves; Tables S8–S10: List of peptides identified with AtRTD2, AtRTD3 and AtRTD3_AtONT-DRS, respectively; Table S11: List of redundant accessions in each protein database; Data S1: AtONT-DRS files; Data S2: AtRTD2 files; Data S3: AtRTD3 files; Data S4: AtRTD3_AtONT-DRS files.
